# Effects of common full-sib families on accuracy of genomic prediction for tagging weight in striped catfish *Pangasianodon hypophthalmus*

**DOI:** 10.3389/fgene.2022.1081246

**Published:** 2023-01-04

**Authors:** Nguyen Thanh Vu, Tran Huu Phuc, Nguyen Hong Nguyen, Nguyen Van Sang

**Affiliations:** ^1^ School of Science, Technology and Engineering, University of the Sunshine Coast, Sippy Downs, QLD, Australia; ^2^ Center for Bio-Innovation, University of the Sunshine Coast, Maroochydore, QLD, Australia; ^3^ Research Institute for Aquaculture No. 2, Ho Chi Minh City, Vietnam

**Keywords:** genetic improvement, genomic selection, growth traits, non-additive genetic estimates and accuracy of selection response, genetic lines

## Abstract

Common full-sib families (*c* ^*2*^) make up a substantial proportion of total phenotypic variation in traits of commercial importance in aquaculture species and omission or inclusion of the *c* ^*2*^ resulted in possible changes in genetic parameter estimates and re-ranking of estimated breeding values. However, the impacts of common full-sib families on accuracy of genomic prediction for commercial traits of economic importance are not well known in many species, including aquatic animals. This research explored the impacts of common full-sib families on accuracy of genomic prediction for tagging weight in a population of striped catfish comprising 11,918 fish traced back to the base population (four generations), in which 560 individuals had genotype records of 14,154 SNPs. Our single step genomic best linear unbiased prediction (ssGLBUP) showed that the accuracy of genomic prediction for tagging weight was reduced by 96.5%–130.3% when the common full-sib families were included in statistical models. The reduction in the prediction accuracy was to a smaller extent in multivariate analysis than in univariate models. Imputation of missing genotypes somewhat reduced the upward biases in the prediction accuracy for tagging weight. It is therefore suggested that genomic evaluation models for traits recorded during the early phase of growth development should account for the common full-sib families to minimise possible biases in the accuracy of genomic prediction and hence, selection response.

## 1 Introduction

In aquaculture species, common full-sib families (*c* ^*2*^) are a result of separate family rearing of about one to 3 months until larvae reach a suitable size for physical tagging (e.g., 10–20 g in fish or 2–5 g in shrimps). The *c* ^*2*^, also known as non-additive genetic components, include both common environmental and maternal effects or possibly dominance, accounting for a significant proportion of total phenotypic variations, ranging from 5% to 55% for growth-related traits in fish (Hamzah et al., 2017; Vu et al., 2019b; Bosworth et al., 2020), crustacean (Nguyen et al., 2020a; Sang N. V. et al., 2020), and mollusc (Sang V. V. et al., 2020). A meta-analysis of 45 studies available in the literature across aquaculture species showed that the mean *c* ^*2*^ value is about 10% for harvest body weight (Nguyen, 2021). Omission of the *c* ^*2*^ resulted in overestimation of heritability by 9%–45% in red tilapia *Oreochromis* spp. (Nguyen et al., 2017; Sukhavachana et al., 2019) or giant freshwater prawn *Macrobrachium rosenbergii* (Luan et al., 2012; Phuc et al., 2021). The estimates of common full-sib families were substantially larger for traits recorded during the early stage of growth development than those measured at harvest. For example, the *c* ^*2*^ values were estimated at .37 for tagging weight vs. .21 for harvest body weight in striped catfish *Pangasianodon hypophthalmus* (Vu et al., 2019b). However, to date, the impacts of common full-sib families on genomic prediction accuracy have not been reported in any aquaculture species, including striped catfish *P. hypophthalmus.*

Current genomic evaluation models used to analyse traits of commercial importance in aquaculture species include only genomic and phenotypic data or combined with pedigree information (e.g., single-step GBLUP). Under these models, the prediction accuracies for body traits (e.g., weight, length) at harvest were moderate to high, ranging from .38 to .89 (Houston et al., 2020). The prediction accuracies for early growth were .33 in common carp *Cyprinus carpio* (Palaiokostas et al., 2018) and .67 for Pacific oysters *Magellana gigas* (Gutierrez et al., 2018). The prediction accuracies for meat quality traits fall within a range of .59–.62 for raw and cooked colour of banana shrimp *Fenneropenaeus merguiensis* (Nguyen et al., 2020b) and .19–.20 for fillet yield and firmness in rainbow trout *Oncorhynchus mykiss* (Al-Tobasei et al., 2021). To date, there is no or limited published information regarding the utilisation of genomic data to assess predictive performance of any statistical methods for tagging weight (i.e., early growth trait) in important aquaculture species.

Almost all studies in aquaculture have employed genomic best linear unbiased prediction (GBLUP), single step- GBLUP (ssGBLUP) or Bayesian methods (Allal and Nguyen, 2022). The Bayesian methods provide flexibility to model different variance distributions of SNPs and can outperform BLUP method (e.g., GBLUP or ssGBLUP) especially for traits under control by genes with large and moderate effects (van den Berg et al., 2015). However, computation of Bayesian methods is highly demanding, and hence, BLUP-family methods have been widely used in practical breeding programs, especially for traits whose variation is of polygenic nature due to many genes, each with very small effects. Recent studies have employed machine and deep learning and obtained higher accuracies for a range of traits than linear (GBLUP) and non-linear Bayesian methods (Yin et al., 2020; Montesinos-López et al., 2021). Regardless of statistical methods used, imputation of missing genotypes or imputation from a low to high density SNP arrays or from commercial SNP arrays to whole genome sequence improved the prediction accuracy for complex traits (Kjetså et al., 2020). Multivariate analysis also slightly increased the prediction accuracy for grain yield in wheat *Triticum aestivum L.* (Sandhu et al., 2021) or cassava *Manihot esculenta* Crantz (Okeke et al., 2017), although its benefits depend on statistical models used (Montesinos-López et al., 2020) or characteristics of datasets and specifically genetic architecture of traits (Gianola and Fernando, 2020). Recent studies have also reported advantages of including functional variants identified from genome-wide associations analysis (GWAS) in prediction models to improve the accuracy of genomic estimated breeding values for growth traits under chronic thermal stress in rainbow trout *O. mykiss* (Yoshida and Yáñez, 2021). In this regard, published information is not available for tagging weight, especially in striped catfish—an important aquaculture species that contributes significantly to the national economies of many countries in Asia, such as Bangladesh, Malaysia, Thailand, Vietnam.

Therefore, this study was set out to test three major hypotheses: 1) omission of the common full-sib families (*c* ^*2*^) from statistical models can result in upward biases in genomic prediction accuracy for tagging weight, 2) imputation of missing genotypes can improve the predictive performance of ssGBLUP, and 3) multi-trait genomic evaluation can lessen the overestimation of the prediction accuracy when the common full-sib families were omitted. Ultimately, the study attempted to explore possibilities for the application of genomic selection for early growth traits in striped catfish.

## 2 Materials and methods

### 2.1 Source of genetic materials

This study included 11,918 fish, which are offspring of 434 females and 278 males in a full pedigree traced back to the base population. The experimental fish were produced between 2015 and 2020, following a semi-nested mating design with a ratio of one male to one or two females (Van Sang et al., 2012). Induced breeding was practised using HCG (Human Chorionic Gonadotropin) hormone with 4 doses (300, 600, 1,200, and 3500 UI). Also note that there are different induction practices regarding doses and types of hormones used, e.g., HCG (Bui et al., 2010) or Suprefact (Samorn, 2007). After striping and ferilizing, eggs were incubated in net jars mounted in a 5 m^3^ composite tank. After hatching, fry of each family was reared in a separate fibreglass tank (1.5 m^3^) for about 3 weeks. Then a random sample of about 500 fry per family were transferred to a net hapa installed in earthen ponds to raise to fingerling size of about 20 g for physical tagging, using Passive Integrated Transponder (PIT). One family was kept in a single hapa net. Three feeding strategies were applied for different rearing periods: no feeding before hatching (0–24 h), Artemia (day 1 to day 3) and Moina (day 4 to day 7) together with fish flake (day 8 to day 15, the foods were made of small size before feeding) were used for tank rearing period before 15 days (Vu et al., 2019b) at a maximum fish uptake and only pellet feed was used during 2 months rearing in earthen pond at a rate of 5% fish biomass. The water was exchanged 50% daily when fish were kept in tank and once per week in pond. In each generation, approximately 200 fish were randomly sampled from each family for PIT tagging. And a half of each family was used for growth testing in the mainstream selection program for increased harvest body weight (Vu et al., 2019b) and another half was sent to concrete tanks for pathogen challenge test to select for increased resistance to *Edwardsiella ictaluri*, a bacterial disease that has caused severe mortality loss during larval and fingerling rearing stages in striped catfish (Vu et al., 2019a). Due to the high mortalities observed after tagging and conditioning, there was a smaller number of fish per family retained for the main challenge test, around 27 fish/family (Table 1).

**TABLE 1:** Descriptive statistics for tagging weight of striped catfish data over 2 generations.

Index	G0-resistance	G1-resistance	G1-growth	All generations
Observation	4937	5224	1757	11918
Number of fish per family	27.6 (10–86)	31.3 (13–87)	18.9 (11–72)	27.1 (10–87)
Weight (g)	23.9 ± 11.7	20.8 ± 11.7	25.0 ± 15.2	22.7 ± 12.4
CV of weight (%)	48.8	55.9	60.7	54.4
Age in day (min—max)	195.0 (149–208)	148.6 (132–180)	178.6 (132–222)	172.2 (132–222)
No. batches	9	4	7	19
No. of sire	107	99	72	272
No. of dam	177	167	90	428
No. of families	179	167	93	439

### 2.2 Trait(s) studied

At tagging, weight of individual fish was recorded using a digital scale with a precision to .1 g. In 2015, 4,937 fish had tagging weight and in the latest generation in 2020, the number of fish with tag weight involved in the pathogen challenge experiment and growth performance testing were 5,224 and 1,757, respectively. In total, there were 11,918 individual data records used in our statistical analysis to assess genomic prediction accuracies. However, due to our limited funding, only a random sample of 560 fish from 40 families in the latest generation (2020) was sequenced to obtain genotype data for our analysis in this study.

### 2.3 Genotype

DNA samples of 560 fish (offspring of 40 females and 31 males) were sent to a commercial service provider in Canberra, Australia for genotyping by sequencing, using Diversity Arrays Technology (DArTseq™). DArTseq™ represents a combination of genome complexity reduction methods and high throughput sequencing platforms (Kilian et al., 2012). A detailed description regarding selections of restricted enzymes, PCR reactions, library preparations and sequencing is given in our earlier studies (Nguyen et al., 2018a; Nguyen et al., 2018b; Nguyen et al., 2020b; Vu et al., 2020). Briefly, sequences generated from each lane were processed using proprietary DArTseq pipelines. Approximately 2,000,000 sequences per barcode/sample were identified and used for variant (SNP) calling. SNP calling was conducted in the DArTsoft14, using DART PL’s C++ algorithm. Calling quality was assured by high average read depth (averaging 60 reads per locus). Furthermore, when multiple polymorphisms were detected on DNA fragments (mostly 75 bp), a single SNP was randomly chosen to avoid linkage disequilibrium between loci. After quality control (QC), we obtained 14,154 SNPs across 560 samples.

### 2.4 Statistical analysis

The missing genotypes (about 10.0%) were imputed using AlphaFamImpute (involving 560 individuals fish and 14,154 SNPs) which takes into account of the pedigree relationships (Whalen et al., 2020). Single-step genomic best linear unbiased prediction (ssGBLUP) method was used to assess genomic prediction accuracy for tag weight. The linear mixed model is written in a matrix notation as follows:

y=Xb+Zu+Wc+e (1)

where

***y*** is the observations related to individual records of each fish.

***X*** is the design matrix related to fixed estimates (***b***) that included generation (1–3) and spawning batch. Age from birth to tagging was also fitted as a linear covariate.

***Z*** and ***W*** are the design matrices related to the additive genetic effects ***u ∼ (0, Hσ*** ^***2***^ ***g*** *)* and common full-sib groups ***c* ∼ *(0, Iσ*** ^***2***^ ***c*** ***)***. The random terms fitted in the model were the additive genetics of individual fish and the common full-sib families. LogLikelihood Ratio Test (LRT) showed that the common full-sib effects were statistically significant for tag weight (Chi-square value with one degree of freedom ranged from 2.3 to 6.2, *p* < .05 to .001). ***e* ∼ *(0, Iσ*** ^***2***^ ***e*** ***)*** is the error term in the model. Where ***I*** is the indentity matrix, ***H*** is described as below. ***σ*** ^***2***^ ***g*** ***, σ*** ^***2***^ ***c*** ***, σ*** ^***2***^ ***e*** are corresponding genetic, common environmental and residual variances.

Our ssGBLUP analysis was conducted in AIREMLf90 of the BLUPF90 package (Misztal et al., 2018). The ssGBLUP is an advanced version of GBLUP that blended numerator relationship (***A***) and kinship (***G***) matrices into a realised ***H*** matrix (Eq. 2), where ***A*** was calculated from the pedigree and ***G*** was computed from 14,154 SNPs. ssGBLUP uses the blended matrix combining both pedigree information and genotype data and hence, is deemed more powerful than GBLUP.

$$H^{-1}=A^{-1}+\left[\begin{array}{cc} 0 & 0\\ 0 & G^{-1}-A_{22}^{-1} \end{array}\right]$$ (2)

The model for single step GWAS expressed as below [also see Aguilar et al. (2019)]:

$$y=Xb+Z_{i}a_{i}+u+e$$ (3)

where *Z*_*i*_ is a vector of SNP values (i.e., 0, 1 or 2), *a*_*i*_ is the effect of the *i*th SNP, *u* is the vector of breeding values obtained from single step analysis from Eq. 1. Here,

$$var\left(u\right)=\frac{ZZ^{\prime }}{\sum {\left(p_{i}\left(1-p_{i}\right)\right)}^{2}}=G\sigma_{u}^{2}$$ (4)

with *p*_*i*_ is the frequency of *i*th SNP, $\sigma_{u}^{2}$ and $\sigma_{e}^{2}$ are assumed known and *y, X, b*, *Z, G,* and *e* are described as above. Analysis of ssGWAS was accomplished by three sub-programs, including blupf90 (computation of genomic breeding values), pregsf90 (derivation of the H matrix) and postgsf90 (estimation of the SNP effect, *p*-values and plotting). The pre-selected SNP panels after GWAS analysis were based on a significant probability of less than .00001 for each of the 25 running sets. Finally, analysis of ssGBLUP genomic prediction were performed using only the highly significant SNPs. The model that omitted the common full-sib effects (c^2^) was the same as Model 1, except that the “Wc” term or “full-sibs” effect was not included.

The predictive performance (or prediction accuracy) of ssGBLUP was evaluated using 5-fold cross validation over five replications. This involved the random division of the phenotypic data into 5 subsets (each with 2383–2384 observations). Then the breeding value of one set was predicted based on the data from the other four subsets. In the five fold cross-validation, the process was repeated 5 times and thus, there were 25 runs in total. The genomic prediction accuracy was defined as the correlations between the predicted breeding values and actual phenotypes ( $r_{y,\hat{y}}$ ) divided by the square root of the trait heritability. The trait heritability was estimated using the AIREML algorithm in the AIREMLF90 of the BLUPF90 family package. The method assumed normal distribution of the variance components for the traits studied; they were the observed heritability for the trait studied. The correlations ( $r_{y,\hat{y}}$ ) were determined as the average value after five-fold cross-validation with 5 repetitions. All single trait analyses were performed in AIREMLf90. Regarding the bivariate analysis, tag weight was co-analysed with survival time (i.e., days that the animals were still alive after the challenge test experiment). The bivariate model was also performed in AIREMLf90. In addition, we analysed the two-trait model [tag weight and survival time (Vu et al., 2021)], using Gibb Sampling method in THRGIBBF1f90 (Tsuruta and Misztal, 2006). In both packages, the bivariate model was the same as Eq. 3 above. In the Gibb sampling, we used 200,000/20,000 and 1,000,000/200,000 as total-cycle/burn-in steps for the univariate and bivariate analyses, respectively. After each Gibbs sampling analysis, results obtained from all the samples were visualised using time series plots of postgibbsf90 program (https://masuday.github.io/blupf90_tutorial/vc_gs.html) to define the stability of variances, and only samples displaying stabilised variances were used to calculate heritability and/or genetic parameters. The prediction accuracies obtained from AIREMLf90 were almost identical to those obtained from THRGIBBF1f90. Thus, only the estimates from the latter analysis were presented in this study. Finally, we performed pedigree-based PBLUP analysis and single-step genome-wide association study (GWAS) in combination of ssGBLUP to better understand the predictive capacity of our statistical models used to analyse tag weight. These analyses used AIREMLf90 and THRGIBBF1f90 packages (Misztal et al., 2018).

Finally, we calculated correlation of EBV for tagging weight between the two statistical models (with and without the common full-sib families) to examine re-ranking effects, i.e., re-ranking of breeding candidates based on their EBVs for tagging weight in the selection program for striped catfish.

## 3 Results

### 3.1 Trait characteristics

The average tag weight of the population was 22.7 ± 12.4 g (Table 1). The tag weight in the first generation (G1, produced in 2019) was slightly lower than that of the base population (G0, produced in 2015) as the animals were tagged at an earlier age (149 vs. 195 d). Despite our efforts to produce all families within a short period in G1 (4–7 spawning batches), the coefficient of variation in the tag weight was somewhat greater in this generation than in the base population (55.9%–60.7% vs. 48.8%). Note that only the animals of generation 1 (560 individuals) had genome sequence and genotype (SNPs) data. The average tag weight of these animals was 23.2 ± 13.0 g.

The heritability (*h* ^*2*^) for tag weight was high (.72–.74) when the common full-sib estimate (*c* ^*2*^) was omitted from our models: PBLUP, ssGBLUP and ssGWAS (Supplementary Table S1). The *h* ^*2*^ estimate obtained from the full models that also included the *c* ^*2*^ estimate was reduced to .15, .08, and .14 for PBLUP, ssGBLUP, and ssGWAS, respectively. The corresponding c^*2*^ estimates were .71, .74, and .72 (Supplementary Table S1).

### 3.2 Accuracy of genomic prediction with and without common full-sib effect (*c* ^*2*^)

**FIGURE 1:**
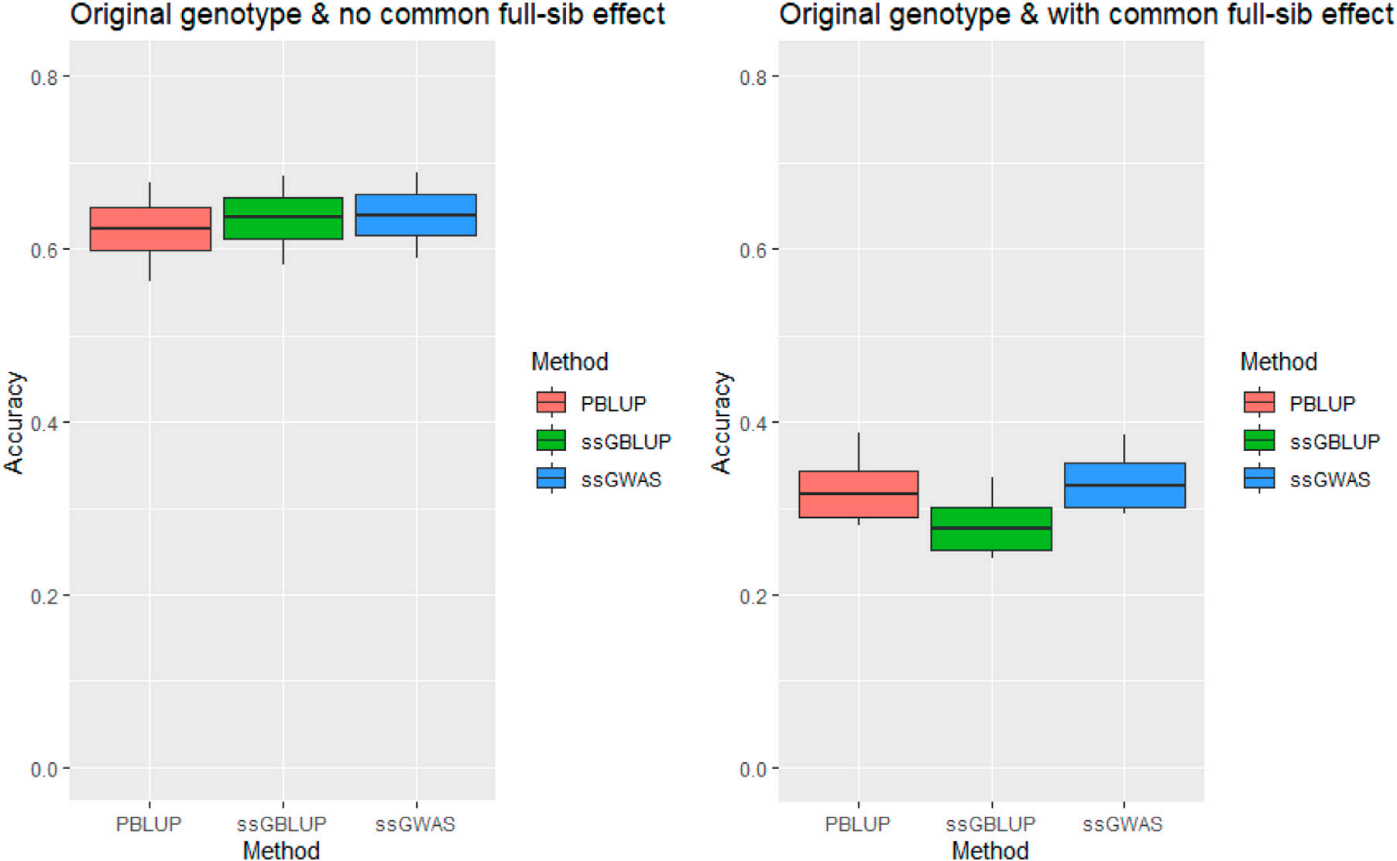
Accuracy of genomic prediction for tagging weight without/with common full-sib effect (*c* ^*2*^) using original genotype under AI-REML algorithm. Middle line of the box is mean accuracy; top and bottom lines of the box is accuracy ± one standard deviation. End points of vertical line represent minimum and maximum values.

The genomic prediction accuracy for tag weight was high (.636) when the *c* ^*2*^ estimates were omitted from our statistical model. However, the accuracy was significantly reduced to .276 in the ssGBLUP model that also included the *c* ^*2*^ estimates (Figure 1). In other words, omission of the *c* ^*2*^ resulted in loss of the prediction accuracy by .278–.334 (or 80.7–105.3%).

### 3.3 Original vs. imputed data using the full model

**FIGURE 2:**
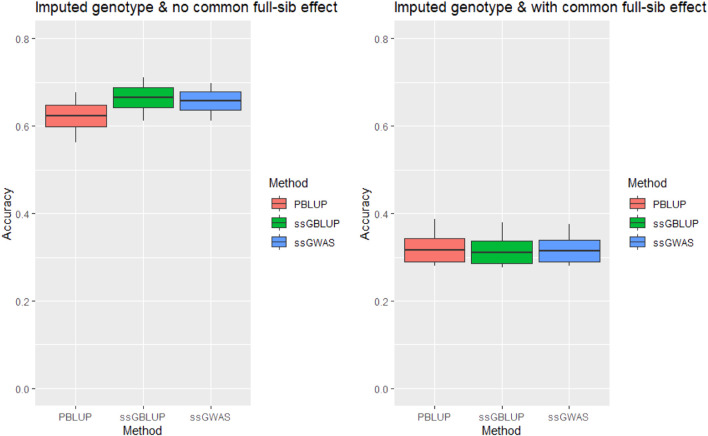
Accuracy of genomic prediction for tagging weight without/with common full-sib effect (*c* ^*2*^) using imputed genotype under AI-REML algorithm. Middle line of the box is mean accuracy; top and bottom lines of the box is accuracy ± one standard deviation. End points of vertical line represent minimum and maximum values.

Imputation of missing genotypes alleviated the upward bias in the prediction accuracy for tag weight when the *c* ^*2*^ estimates were fitted in statistical models of our analysis (Figure 2). The accuracy obtained from the full ssGBLUP model that included the *c* ^*2*^ estimates was .311 when the imputed genotype was analysed as compared with .276 of the original data. This means that imputation improved the prediction accuracy for tag weight by .035 (or 12.8%) (also see Supplementary Table S2).

### 3.4 Multi-trait analysis using the full model

Bivariate analysis involving tag weight and a disease resistance trait (i.e., survival time) improved the prediction accuracy by .031 (or 11.2%) relative to the univariate ssGBLUP model (.307 vs. .276). The two-trait analysis also reduced biases in the prediction accuracy for tag weight when the *c* ^*2*^ were included in our models (*r* = .3098 for the two-trait model with the *c* ^*2*^ estimates vs. .630 without the *c* ^*2*^). Regardless of the inclusion or exclusion of the *c* ^*2*^, the prediction accuracies were similar between AI-REML and Gibb sampling methods either when original genotypes (Table 2) or imputed genotypes (Table 3) were analysed.

**TABLE 2:** Genomic prediction accuracy from multivariate models in AIREMLf90 and THRGIBBS1f90, using original (un-imputed) genotypes.

Method	AIREMLf90			THRGIBBS1f90		
	Without *c* ^*2*^	With *c* ^*2*^	Difference (%)	Without *c* ^*2*^	With *c* ^*2*^	Difference (%)
PBLUP	.6272 ± .025	.3471 ± .025	80.7	.6227 ± .025	.3446 ± .025	80.7
ssGBLUP	.6371 ± .023	.3067 ± .023	107.7	.6360 ± .023	.3098 ± .022	105.3
ssGWAS	.6391 ± .023	.3264 ± .026	95.8	.6392 ± .023	.3451 ± .026	85.2

**TABLE 3:** Genomic prediction accuracy from multivariate models in AIREMLf90 and THRGIBBS1f90, using imputed genotypes.

Method	AIREMLf90			THRGIBBS1f90		
	Without *c* ^*2*^	With *c* ^*2*^	Difference (%)	Without *c* ^*2*^	With *c* ^*2*^	Difference (%)
PBLUP	.6272 ± .025	.3471 ± .025	80.7	.6227 ± .025	.3446 ± .025	80.7
ssGBLUP	.6644 ± .023	.3323 ± .026	99.9	.6658 ± .023	.3317 ± .024	100.7
ssGWAS	.6571 ± .022	.3352 ± .023	96.0	.6578 ± .021	.3331 ± .021	97.5

### 3.5 ssGWAS in combination with ssGBLUP

The inclusion of highly significant markers (471 SNPs) slightly increased the prediction accuracy for tag weight relative to ssGBLUP (Table 2). However, it had little impacts on the upward biases in the prediction accuracy when the common full-sib families were omitted from our univariate (Figures 1, 2) and multi-variate analyses (Table 3), using either linear mixed model or threshold Gibb sampling methods.

### 3.6 Re-ranking effects

To examine the impact of the common full-sib families on re-ranking effects, we calculated correlation of EBV for tagging weight between the two models (with the presence and absence of the common full-sibs effect). The Pearson correlation coefficient ranged from .30 to .62 (Supplementary Table S3), suggesting potential re-ranking effects of selection candidates when the c^2^ effects were not included in genomic evaluation models for tagging weight of striped catfish.

## 4 Discussion

In the present study we attempted to address five major questions which are worth considering before initiating genomic selection program for early growth in striped catfish as well as other aquaculture species of economic importance.

### 4.1 Should genomic selection be practised for tagging weight?

The prediction accuracy for tagging weight was high due to the high heritability (.72–.74) for this trait, which is opening new opportunities for improving early growth through genomic selection. Selection for early growth could shorten generation time of striped catfish *P. hypophthalmus* which often takes 3–4 years to maintain a breeding cycle in genetic improvement programs. However, selection for tag weight may not capture all genetic variation in body traits at harvest as the genetic correlation (*r*_*g*_) between these two traits is reported to be .5 in this population (Vu et al., 2019b). In Asian seabass *Lates calcarifer*, Khang et al. (2018) also observed a significantly different from one genetic correlation (*r*_*g*_ = .31–.47) for body weights between successive rearing periods from 180 to 556 days post-hatch. Based on the genetic correlation estimates between tag and harvest weights, it is necessary to examine genomic prediction accuracy for harvest weight in this population of striped catfish. Furthermore, there are also no clear advantages regarding the prediction accuracy of ssGBLUP and ssGWAS as compared with PBLUP in our study. Future work should consider enlarging the sample size (in terms of the number of individuals and families genotyped) and number of SNPs to take the advantages of ssGBLUP and ssGWAS models that can capture some measures of Mendelian sampling to improve the estimation of genetic (kinship) matrices for all individuals in the pedigree and hence, improving accuracy of estimated breeding values for tagging weight in this population of striped catfish.

### 4.2 Does omission of the common full-sib effects affect the genomic prediction accuracy?

When the common full-sib families (*c* ^*2*^) were excluded from our statistical methods, this resulted in upward biases in the prediction accuracy by 96.5%–130.3% for tagging weight. The overestimation of the prediction accuracy was to a greater extent when PBLUP was used as compared with other methods (i.e., GBLUP and ssGBLUP). To date, no published information is available in aquaculture species to compare with our studies. However, studies in farmed animals suggested that effects of non-additive genetics should be included in mating structures to improve accuracy of genomic prediction and hence, maximizing productivity for dairy farms (Aliloo et al., 2017; Varona et al., 2018). Conventional genetic evaluation systems using pedigree and phenotype data in aquaculture species have also shown that the animal breeding values (EBVs) estimated for growth traits were overestimated, for instance, 10%–56% in giant freshwater prawn *M. rosenbergii* (Phuc et al., 2021) or red tilapia *O.* spp. (Nguyen et al., 2017). Hence, our results are as expected because the *c* ^*2*^ estimates were often large for growth traits in aquaculture species where separate rearing of each family was often conducted over a period of 2–3 months until the fish reach a suitable size (e.g., 10–20 g) for physical tagging. The *c* ^*2*^ estimates were generally not significant if early communal rearing of all families is practised and DNA markers are used for parentage assignment, as demonstrated in common carp *C. carpio* (Ninh et al., 2013) or yellowtail kingfish *S. lalandi* (Premachandra et al., 2017). Collectively, due to the high c^2^ effects on tagging weight and its low to moderate genetic correlation with market (harvest) weight, genomic evaluation models for these traits should account for the common full-sib families and they should be considered as separate traits in genetic improvement programs for striped catfish as well as other aquaculture species.

### 4.3 Can multivariate analysis lessen the upward biases in the prediction accuracy?

Our multivariate analysis of tagging weight in combination with disease resistance trait (survival time) aimed to utilise genetic covariation between the traits and hence improved the predictive power of statistical models used. In addition, when the *c* ^*2*^ were omitted, the extent of the overestimation in the prediction accuracy was smaller in the multivariate analysis than univariate models. Studies in animals and plants have reported that multi-trait analysis can improve the prediction accuracy for productivity traits (e.g., milk yield in cattle or grain yield in wheat *T. aestivum L.*) by 0%–28.5% (Sandhu et al., 2021). However, other studies also showed that there are little or no benefits of multivariate vs. single trait analysis (Kemper et al., 2018). To date, studies in aquaculture species performed multi-trait genomic prediction are limited. Results from these studies showed that the accuracies of genomic predictions were not improved for fillet weight and fillet yield in Nile tilapia *O. niloticus* (Joshi et al., 2020) or for survival status and survival time in striped catfish *P. hypophthalmus* (Vu et al., 2021), likely because the high heritability of these two traits and their high genetic correlations; hence, adding one trait did not improve the prediction accuracy of the other. In yellowtail kingfish, Nguyen et al. (2022) also showed that the benefits of multi- vs. univariate analysis depend on statistical methods used and genomic architecture of traits. Hence, molecular dissection of the genomic architecture of traits (e.g., identifying pleotropic loci) can help further understand the impacts of multi-trait analysis on the prediction accuracy for tagging weight and disease resistance examined in this population.

### 4.4 What can imputation help in genomic prediction?

In this study, we found that imputation of missing genotypes has two major benefits. First, it improved the prediction accuracy for tagging weight by 2.1%–12.8%, as compared with when the original (un-imputed) data were used. Second, the imputation reduced the upward biases in the prediction accuracy for tagging weight when the *c* ^*2*^ estimates were omitted from our statistical models, mainly because the complete genotypes improved accuracy of estimated breeding values for tagging weight. The benefit of imputation on genomic prediction in aquaculture breeding has been reported in recent studies, such as for disease resistance to photobacteriosis in gilthead sea bream *S. aurata* (Bargelloni et al., 2021), resistance to sea lice in Atlantic salmon *S. salar* (Tsai et al., 2017; Kjetså et al., 2020), growth-related traits Yellowtail kingfish *Seriola lalandi* (Nguyen et al., 2018a) or with simulated data in rainbow trout *O. mykiss* (Dufflocq et al., 2019). In selective breeding programs, imputation can help to reduce costs associated with sequencing. One option is to perform low-density genome sequence (Kriaridou et al., 2020) for a large number of selection candidates and high-density sequence for only parents (Tsai et al., 2017). Then imputation is made to impute from low to high or whole genome sequence. This would help increase selection intensity and thus genetic gain made in selected populations. Furthermore, when more data are accumulated in this population, imputation can increase power of detecting variants for tag weight in genome-wide association studies or fine mapping analysis, integrate multi-studies for meta-analysis of datasets, which are genotyped on different platforms or level of genome coverage. However, also note that the performance of genotype or sequence imputation is affected by many factors, such as reference selection, SNP density, sample size, sequence coverage, minor allele frequency of populations (Chen et al., 2014; Druet et al., 2014; Dufflocq et al., 2019). These factors are fully or partially accounted for in recent software packages that can facilitate the imputation in our breeding program for high growth in striped catfish *P. hypophthalmus*.

### 4.5 Can ssGWAS alleviate the impacts of the c^2^ omission on the prediction accuracy?

Inclusion of highly significant SNPs in genomic prediction models that included the *c* ^*2*^ did not have noticeable impacts on the prediction accuracy for tagging weight. This is likely due to the limited size of the significant SNPs obtained from genotyping by sequencing (GBS) platform but our observation here is consistent with previous findings for disease resistance traits in the same population of striped catfish *P. hypophthalmus* (Vu et al., 2021). In studies where the *c* ^*2*^ estimates were not included, Luo et al. (2021) also found there were no advantages of pre-selected SNPs in genomic prediction models using ssGBLUP, WssGBLUP and BayesB for resistance to *Edwardsiella tarda* that causes acute symptoms with ascites in Japanese flounder (*Paralichthys olivaceus*). However, other studies, which used prioritised variants from GWAS, reported there was an improvement in the prediction accuracy by 1.2%–13.3% for growth-related traits under chronic thermal stress in rainbow trout *O. mykiss* (Yoshida and Yáñez, 2021) or disease resistance traits in whiteleg shrimp *L. vannamei*, Atlantic salmon *S. salar* and gilthead sea bream *S. aurata* (Luo et al., 2021). In addition, the variant (or marker) effects can be weighed to improve the prediction accuracy as demonstrated in our recent study for disease traits (Vu et al., 2021) or for production traits in dairy cattle (Xiang et al., 2021).

## 5 Concluding remarks

The prediction accuracy for tagging weight using BLUP-family methods was moderate to high. The omission of the common full-sib families resulted in upward biases in the predictive performance across statistical models used. Imputation of missing values alleviated the impacts of the common full-sib families on the prediction accuracy. As compared with single trait analysis, multivariate model slightly improved the prediction accuracy when the *c* ^*2*^ effects were excluded from our analyses. A combined ssGWAS with ssGBLUP did not sacrifice the prediction accuracy, regardless of the *c* ^*2*^. Our results suggest that genomic selection for early growth traits should include the *c* ^*2*^ in statistical models to investigate any possible changes in selection accuracy and selection response. Future study should increase the number of genotyped individuals and/or consider alternative genotyping platforms (e.g., whole genome sequencing) as well as use different mating structures (e.g., using full or partial factorial design) to enable the separation of the dominance from common full-sib effects in order to improve accuracy of genomic prediction for tagging weight and commercial traits of economic importance in this striped catfish population.

## Data Availability

The original contributions presented in the study are included in the article/Supplementary Material, further inquiries can be directed to the corresponding authors.
