# Supplementary material for: Effects of common full-sib families on accuracy of genomic prediction for tagging weight in striped catfish Pangasianodon hypophthalmus
Source: Front Genet. 2023 Jan 4;13:1081246. doi: 10.3389/fgene.2022.1081246 (PMC9845282; doi:10.3389/fgene.2022.1081246)
Supplement: Supplementary file 1 [file Table1.docx]

**Effects of common full-sib families on accuracy of genomic prediction for tagging weight in striped catfish *Pangasianodon hypophthalmus***

Nguyen Thanh Vu^a,b,c^, Tran Huu Phuc^c,1^, Nguyen Hong Nguyen^a,b*^, and Nguyen Van Sang^c,1,*^

^a^School of Science, Technology and Engineering, University of the Sunshine Coast, 90 Sippy Downs drive, Sippy Downs, QLD 4556, Australia

^b^Center for Bio-innovation, University of the Sunshine Coast, Australia

^c^Research Institute for Aquaculture No.2, 116 Nguyen Dinh Chieu Street, District 1, Ho Chi Minh city, Vietnam

^1^Equal contribution

*Correspondence to N.H. Nguyen [nnguyen@usc.edu.au](mailto:nnguyen@usc.edu.au) and N. V. Sang: [sangnv.ria2@mard.gov.vn](mailto:sangnv.ria2@mard.gov.vn)

Supplementary Table S1. Variances components, heritability estimates (h^2^) for tagging weight when the common full-sibs were included or excluded from the statistical models

|  | omission of c^2^ | | | inclusion of c^2^ | | | | | |
| --- | --- | --- | --- | --- | --- | --- | --- | --- | --- |
| Method | $\sigma_{a}^{2}$ | $\sigma_{e}^{2}$ | h^2^ ± se | $\sigma_{a}^{2}$ | $\sigma_{c}^{2}$ | $\sigma_{e}^{2}$ | h^2^ ± se | c^2^ ± se |  |
| PBLUP | 52.1 | 19.8 | 0.72±0.01 | 22.0 | 105.2 | 20.8 | 0.15±0.01 | 0.71± 0.03 |  |
| ssGBLUP | 53.0 | 19.1 | 0.74±0.01 | 12.0 | 109.1 | 25.8 | 0.08±0.02 | 0.74± 0.03 |  |
| ssGWAS | 52.0 | 19.9 | 0.72±0.01 | 19.9 | 105.4 | 21.8 | 0.14±0.04 | 0.72±0.02 |  |

$\sigma_{a}^{2},$ $\sigma_{c}^{2}$, $\sigma_{e}^{2}$are genetic , common environmental and residual variances

Supplementary Table S2. Prediction accuracy for tagging weight when the common full-sib families (*c^2^*) were included or excluded in univariate model of AIREMLf90 using original (un-imputed) and imputed genotypes.

|  | Original genotype | | | Imputed genotype | | |
| --- | --- | --- | --- | --- | --- | --- |
| Method | Without *c^2^* | With *c^2^* | Difference (%) | Without *c^2^* | With *c^2^* | Difference (%) |
| PBLUP | 0.6232 ± 0.025 | 0.3172 ± 0.027 | 96.5 | n.a. | n.a. |  |
| ssGBLUP | 0.6359 ± 0.024 | 0.2761 ± 0.025 | 130.3 | 0.6359 ± 0.024 | 0.3114 ± 0.027 | 104.2 |
| ssGWAS | 0.6575 ± 0.022 | 0.3264 ± 0.026 | 101.4 | 0.6575 ± 0.022 | 0.3114 ± 0.034 | 111.1 |

n.a. = not available (PBLUP uses phenotype and pedigree information only)

Supplementary Table S3: Correlation of estimated breeding values for tag weight between the two statistical models (with and without c^2^).

| *Methods* | *BLUP_ without* c^2^ | *ssGBLUP_ without* c^2^ | *ssGWAS_ without* c^2^ | *BLUPwith* c^2^ | *ssGBLUP with_* c^2^ |
| --- | --- | --- | --- | --- | --- |
| ssGBLUP_ *without* c^2^ | 0.793 |  |  |  |  |
| ssGWAS_ *without* c^2^ | 0.776 | 0.827 |  |  |  |
| BLUPwith c^2^ | 0.615 | 0.318 | 0.454 |  |  |
| ssGBLUP with_ c^2^ | 0.464 | 0.295 | 0.364 | 0.863 |  |
| ssGWAS with_ c^2^ | 0.464 | 0.295 | 0.364 | 0.863 | 1.000 |
